# An analysis of clinical activity, admission rates, length of hospital stay, and economic impact after a temporary loss of 50% of the non-operative podiatrists from a tertiary specialist foot clinic in the United Kingdom

**DOI:** 10.3402/dfa.v4i0.21757

**Published:** 2013-09-10

**Authors:** Catherine Gooday, Rachel Murchison, Ketan Dhatariya

**Affiliations:** Diabetic Foot Clinic, Elsie Bertram Diabetes Centre, Norfolk and Norwich University Hospitals National Health Service Foundation Trust, Norwich, Norfolk, UK

**Keywords:** diabetes, foot clinic, podiatrist, economic value, multidisciplinary team

## Abstract

**Introduction:**

Podiatrists form an integral part of the multidisciplinary foot team in the treatment of diabetic foot–related complications. A set of unforeseen circumstances within our specialist diabetes foot service in the United Kingdom caused a loss of 50% of our non-operative podiatry team for almost 7 months during 2010. Some of this time was filled by non-specialist community non-operative podiatrists.

**Methods:**

We assessed the economic impact of this loss by examining data for the 5 years prior to this 7-month interruption, and for the 2 years after ‘normal service’ was resumed.

**Results:**

Our data show that the loss of the non-operative podiatrists led to a significant rise in the numbers of admissions into hospital, and hospital length of stay also increased. At our institution a single bed day cost is £275. During the time that the numbers of specialist non-operative podiatry staff were depleted, and for up to 6 months after they returned to normal activities, the extra costs increased by just less than £90,000. The number of people admitted directly from specialist vascular and orthopaedic clinics is likely to have increased due to the lack of capacity to manage them in the diabetic foot clinic. Our data were unable to assess these individuals and did not look at the costs saved from avoiding surgery. Thus the actual costs incurred are likely to be higher.

**Conclusions:**

Our data suggest that specialist non-operative podiatrists involved in the treatment of the diabetic foot may prevent unwarranted hospital admission and increased hospitalisation rates by providing skilled assessment and care in the outpatient clinical settings.

Diabetes related foot disease remains one of the most frequent causes of ‘diabetes specific’ hospital admissions (1). In England alone there are estimated to be 6,000 diabetes related amputations per year, with the estimated cost of diabetes related foot disease – in particular ulceration and amputation – calculated to be between £639 and £662 million per annum (2, 3). A recent economic analysis of health spending in diabetes in England stated that approximately £1 in every £150 spent by the National Health Service (NHS) in England each year is on diabetes related foot disease (3).

In 1986, a seminal paper from the team at King's College Hospital, London, demonstrated a higher rate of ulcer healing and decreased numbers of major amputations after the introduction of a dedicated diabetic foot clinic (DFC) which gathered a multidisciplinary team (MDT), including the services of podiatrists, together in one place (4).

Podiatrists are often the frontline professionals in the MDT for the treatment of diabetic foot related complications. The UK National Diabetes Inpatient Audit 2010 demonstrated that specialist podiatrists were the largest professional group represented in the MDT, with 78% of teams having one (1). Their importance was further highlighted by a meeting of the UK Parliamentary Commons Health Select Committee held in March 2013 which called for sufficient numbers of specialist podiatrist posts to be funded in order to support the diabetic foot MDT (5). However, in the current financial climate, many purchasers of specialist services require economic data to support these arguments. There are recent data from the United States to show the economic value of physicians or surgeons of the foot and ankle (6) in reducing the cost burden of diabetic foot disease, but to date there has not been an economic analysis done to support the benefits of podiatrists, in particular when addressing admission avoidance, reducing length of hospital stay, and reduction in amputation rates.

The DFC in the Norfolk and Norwich University Hospital NHS Foundation Trust is a tertiary centre of excellence for the management of acute diabetic foot complications (7, 8). The hospital has 989 adult beds and serves a population of 700,000, of whom 32,000 have diabetes, across a geographical area of approximately 2,000 square miles. The 3.89 whole time equivalent (WTE) podiatrists triage and assess new referrals, with 40% being seen in the specialist podiatrist outpatient clinic in one working day. The podiatrists have extensive diagnostic and clinical skills in the management of diabetic complications and have the responsibility for the management of the majority of the patients referred to the DFC. Appropriate referrals are also initiated if necessary to the specialist consultant-led medical, vascular, and orthopaedic clinics that are available every 2 weeks in the centre (i.e. six consultant-led specialist clinics per month). Direct hospital admissions from the DFC are also arranged by the podiatrists when they deem it necessary for foot infections, with or without systemic signs (9), and for individuals with critical or acute ischemia. In addition, the podiatrists provide a dedicated daily inpatient service providing care and education for hospitalised patients, those known to the DFC, and those not known but identified with foot problems by the medical and nursing staff. The full MDT (consultants in diabetes, vascular surgery, orthopaedic surgery, and microbiology) also does a weekly ward round.

Within a 6-week period in 2010 specialist podiatry staffing in the DFC was reduced to 1.89 WTE for 7 months. This was a result of two posts being made vacant due to a staff member relocating to a different part of the United Kingdom and another staff member experiencing unexpected long term sickness. Subsequently the capacity of the service was significantly affected, with increased waiting times for new patients and intervals between follow-up appointments for existing patients being extended. Patients assessed as ‘stable’ or ‘close to healing’ had their discharge to the community foot protection team brought forward.

In this paper, the definition of a specialist podiatrist included the healthcare non-operative podiatrist involved in a MDT tertiary foot clinic for the management of the diabetic foot and its related complications. Our goal was to assess the economic impact of a loss of half of our healthcare podiatrists by undertaking a formal review of our activity before and after a 7-month interruption of normal service.

## Methods and materials

At our institution, activity data for the specialist DFC are exported directly from the Patient Administration System and collated on a monthly basis. Admissions to hospital from the DFC are collected prospectively by the podiatrists at the time of admission. This information is stored on a password protected Excel^®^ spreadsheet (Microsoft, Berkshire, UK) and following discharge the length of stay (LOS) is added. Because the data presented in this study were contemporaneously collected by a small number of specialist staff who knew almost all of the patients personally, we were confident that we had a complete data set. However, admissions from other sources collected from clinical coding such as the Accident and Emergency department or Emergency Assessment Units were not recorded in this study because previous work done in our institution has shown inaccuracies in clinical coding and discharge data (10).

We analysed the change in activity levels in two different ways by looking at the impact on inpatient and outpatient activity. First, because in our institution the cost of a ‘hospital bed day’ was estimated at £275, we compared the cost of admissions before and after the 50% reduction in podiatry staffing levels. Second, the DFC currently receives income from the commissioning organisation on a ‘cost and volume’ tariff payment system. The ‘cost’ is based on the complexity of the assessment and treatment required by patients, which is divided into new patients and simple or complex follow-ups. The ‘volume’ is how many patients are seen. These data are shown in Table 1. We analysed how this reduction in activity in 2010 affected the income the DFC received.

**Table 1:** Clinic activity divided into patient complexity

Year	Total contacts	New patients	Total follow-up	Simple follow-up	Complex follow-up	Income (£) (new patients)	Income (£) (simple follow-up)	Income (£) (complex follow-up)	Total income (£)
2008	4,197	344	3,853	3,286	567	48,504	230,020	79,947	358,471
2009	4,799	356	4,443	3,794	649	50,196	265,580	91,509	407,285
**2010**	**4,058**	**382**	**3,676**	**2,932**	**744**	**53,862**	**205,240**	**104,904**	**314,006**
2011	4,294	509	3,785	2,555	1,230	71,769	178,850	173,430	424,049
2012	5,270	697	4,573	3,534	1,039	98,277	247,380	146,499	492,106

Values in bold represent the year of service interruption. The income generated from a new patient is £141; for ‘simple’ follow-up feet, £70; and for ‘complex’ feet, £141. The income generated by the loss of podiatry staff meant a reduction in income from the year before of 23%.

We also compared the number of admissions due to diabetic foot complications and looked at the number of overall ‘bed days’ (i.e. number of days per year that a bed at our institution was occupied by someone with a diabetic foot problem). In addition, we looked at the LOS of hospital inpatients admitted with foot problems before and during the period of staff shortage. In 2010 in the United Kingdom, the annual salary of a full time senior specialist podiatrist was £35,184. Thus we were also able to compare the costs incurred by employing someone to the impact on the overall local health economy of them not being employed (11).

## Results

The activity levels for the clinic between 2005 and 2012 are shown in Table 2. Foot clinic activity steadily increased from 2,835 in 2005 to 5,270 in 2012. To accommodate this increase in clinical activity, podiatry staffing levels in the DFC had remained stable at 3.89 WTE since 2008. During the 5 years before the staff shortages described, the average number of admissions directly from the DFC was 44 per year and the LOS 16.6 days. At our institution, a ‘hospital bed day’ costs £275. The increase in hospital admissions and LOS during the staff shortage equated to 327 extra bed days compared to the 12 months prior to service disruption. The increased expenditure for this year equated to £89,925.

**Table 2:** Clinical activity between 2005 and 2012, showing the drop in number of people seen when the number of staff dropped, but a corresponding increase in the proportion of people admitted, and an increase in their hospital length of stay

Year	Clinic activity	No. of admissions	Admissions as a% of total activity	Total bed days	Mean Length Mean length of hospital stay (±SD)
2005	2,835	30	1	515	17.2 (9.2)
2006	2,921	43	1.5	775	17.2 (19.2)
2007	3,325	39	1.1	570	14.6 (11.3)
2008	4,197	50	1.2	919	18.4 (16.8)
2009	4,799	58	1.2	867	14.7 (11.3)
**2010**	**4,058**	**72**	**1.8**	**1,194**	**16.5 (12.3)**
2011	4,294	41	0.95	838	20.4 (16.6)
2012	5,270	45	0.89	733	16.2 (15.1)

Values in bold represent the year of service interruption.

These data were a powerful argument in releasing resources from the commissioning organisation to allow the vacant post to be advertised and to secure the employment of temporary (non-specialist) podiatrists from the community foot protection team to partially cover one of the vacant posts. However, because the temporary staff were non-specialists, clinic capacity was still not optimised because of the requirement to supervise them. Specialist staffing levels and activity levels were eventually restored more than 7 months after the original loss of staff.

Following staffing and activity levels returning to normal, it took more than a year to restore the number of outpatient patient contacts. As shown in Table 2, the number of patient contacts went down from 4,799 individual contacts in 2009 to 4,058 in 2010, and when the number of podiatrists was restored, it rose eventually to 5,270 in 2012. In addition, it took more than a year to reduce the number of hospital admissions directly from the DFC back to 45 in 2012, which reflected the average of the 5 years preceding the staffing loss.

The number of new patients that was seen in 2009 was 356 and this rose to 382 in 2010. This is in line with the number of new referrals that we felt needed to be seen. This would be partly because of the increased awareness of our clinic service amongst primary care practitioners across the whole of Norfolk and the introduction of our new antibiotic protocol (9), but also because of a number of education initiatives that the podiatry team had put in place. It also reflected the increasing prevalence of diabetes within our population over time.

It can be seen in Table 2 that the total number of follow-ups seen in 2009 dropped by more than 17% in 2010, reflecting the reduction in clinic capacity. The main cause for this reduction was the lack of capacity to see the ‘simple’ follow-ups (i.e. feet that were at the least risk and could be followed up by the community foot protection team). Overall, the number of patients referred to the foot clinic with complex problems increased as shown by the increase in these types of contacts. These became the focus of foot clinic attention during this period of staff shortages.

As a result of the number of people in the complex foot clinic being seen, the income from this category of patient rose from £91,509 to £104,904. However, because the number of ‘simple’ follow-ups reduced substantially, this equated to a decrease in income from this category of patient from £265,580 in 2009 to £205,240 in 2010. As the number of clinic staff rose again there was a corresponding increase in the number of follow-ups that could be seen and in the income generated from our clinic.

## Discussion

The present data show the economic value of the podiatrists in the diabetic foot MDT by helping to reduce acute hospital admissions and reducing hospital LOS. Our data demonstrate that reduced investment in podiatry frontline services and the DFC MDT had a negative impact on outcomes. In this instance, the financial savings associated with not filling the vacant podiatry position and failing to provide adequate resources to cover sickness led to an increase in the overall costs associated with caring for people with acute diabetic foot complications.

There is an ever increasing global prevalence of diabetes and about 347 million people worldwide have the disease (12). In 2012, the prevalence of diabetes in the United Kingdom was estimated to be 5.6% (12). The Association of Public Health Observatories Diabetes Prevalence Model predicts that the number of people with diabetes in England will rise by 23% between 2010 and 2020 (13). Along with the rise in the prevalence of diabetes, the number of people with complications is also expected to rise at the same rate. Diabetes related foot disease remains a major global public health burden and international efforts are being made to ensure that all people with foot disease have access to high quality care when and where they need it (14).

As part of providing good foot care, there is evidence to support the positive impact of the MDT in improving outcomes for patients with acute diabetic foot complications (4). One centre in the United Kingdom showed that following the introduction of a MDT, major amputations rate fell by 82% from 41.4 to 6.7 per 10,000 people with diabetes (15). Another centre showed a reduction in mean LOS for patients with diabetic foot ulcerations from 50 days to 19 days following the introduction of their MDT (16). Across the United Kingdom many guidelines have been introduced supporting this model of care (17–19). However, despite these recommendations and the evidence for their effectiveness, the National Diabetes Audit in 2011 found that 40.5% of hospitals did not have a dedicated, functioning, properly staffed MDT (1).

The MDT is essential for effective management of the acute diabetic foot. When this is disrupted or absent the consequences for patients can be very detrimental. The NHS Atlas of variation showed a sixfold variation in amputation rates across England (20). The data we have presented have demonstrated how a reduction in access to services may potentially be a reason for the variations described. In addition, our data have shown that when specialist staffing levels were adequate, hospital admission rates from the DFC remained consistent. This was despite the rising prevalence of diabetes and the increasing number of new patient referrals to the foot clinic. Thus, as a percentage of total activity, acute hospital admission dropped.

The annual costs of diabetic foot disease to healthcare agencies in the United Kingdom are estimated to exceed £1 billion (3). Increasing and improving access to specialist podiatrists and ensuring they are actively involved in the DFC MDT has the potential to decrease the overall cost associated with managing acute diabetic foot complications.

This paper has only focused on admissions, not the outcome of these admissions, which may potentially have resulted in avoidable amputations. The Healthcare Resource Group tariff in 2010–2011 for a major lower extremity amputation was £9,477 (21). The Department of Health Audit Office estimated that reducing late referrals by 50% could save £34 million a year through reducing amputation rates (22). In order to achieve this saving, we believe that diabetic foot teams should have ready access to the services of specialist diabetes podiatrists who have the necessary knowledge and skills to deal with these complex patients.

Some of the strengths of our data were that we prospectively collected and covered all patients seen or admitted directly from the foot clinic and we could accurately collect their length of hospital stay. There are, however, potential weaknesses to our data. First, we were unable to collect data on all of the patients admitted from the specialist clinics, and this could have suggested that our costs were an underestimate. In addition, the data presented are from only one specialist centre, whereas further work to support our hypothesis would need to come from economic analyses of prospective data collected from other units with reductions in staffing levels. Furthermore, data collected over a longer period of time would potentially be of use.

## Conclusion

This paper from our institution demonstrates that podiatrists in the United Kingdom play an essential and key role within the MDT, often acting as ‘gate keepers’, preventing hospital admission by providing skilled assessment and care in outpatient settings.
